# Translating Community Connectedness to Practice: A Qualitative Study of Midlevel Health Workers in Rural Guatemala

**DOI:** 10.5402/2012/648769

**Published:** 2012-10-14

**Authors:** Alison Hernández, Anna-Karin Hurtig, Kjerstin Dahlblom, Miguel San Sebastián

**Affiliations:** Umeå International School of Public Health, Department of Public Health and Clinical Medicine, Umeå University, 901 87 Umeå, Sweden

## Abstract

*Background*. The performance of midlevel health workers is a critical lever for strengthening health systems and redressing inequalities in underserved areas. Auxiliary nurses form the largest cadre of health workers in Guatemala. In rural settings, they provide essential services to vulnerable communities, and thus have great potential to address priority health needs. This paper examines auxiliary nurses' motivation and satisfaction, and the coping strategies they use to respond to challenges they confront in their practice. *Methods*. Semistructured interviews were conducted with 14 auxiliary nurses delivering health services in Alta Verapaz, Guatemala. *Results*. Community connectedness was central to motivation in this rural Guatemalan setting. Participants were from rural communities and conveyed a sense of connection to the people they were serving through shared culture and their own experiences of health needs. Satisfaction was derived through recognition from the community and a sense of valuing their work. Auxiliary nurses described challenges commonly faced in low-resource settings. Findings indicated they were actively confronting these challenges through their own initiative. *Conclusions*. Strategies to support the performance of midlevel health workers should focus on mechanisms to make training accessible to rural residents, support problem-solving in practice, and emphasize building relationships with communities served.

## 1. Background 

Midlevel health workers (MLHWs) are the mainstay of health service delivery in many low income countries. Given their prominent role, supporting the performance of MLHWs is a critical lever for strengthening health systems and redressing health inequalities in underserved areas. MLHWs are defined as health care providers with formal accreditation but shorter training and a more restricted scope of practice than professionals [1]. MLHW cadres share characteristics that make them particularly attractive for initiatives to improve access to care and mitigate health workforce deficits [2, 3]. Lower entry level education requirements make it easier to recruit locals who have potential to provide more culturally and linguistically appropriate care and may be easier to retain in rural placements [4]. Studies indicate that with adequate training and continued support, MLHWs can provide care comparable to professionals [5]. Increasing deployment of MLHWs can also be appealing as a lower cost alternative for improving coverage. 

Despite their centrality in the health workforce, MLHW cadres tend to have low status and inadequate recognition. They frequently lack professional organizations, and national information systems often do not differentiate them from professionals, making it difficult to monitor their deployment and productivity [6]. These factors contribute to their weak integration into the health system, which is manifested in lack of attention to management issues, training, support, career development, and progression [1, 7]. As they often work in underserved remote areas, many face constraints which affect performance such as staffing shortages, lack of medicine, and equipment. 

The challenging situation that MLHWs and other service providers face exemplifies the complexity of improving performance in resource-constrained settings. Efforts have traditionally focused on enhancing health worker knowledge and skills, but there is increasing recognition that abilities alone are insufficient to achieve improvement in these challenging conditions [8]. The 2006 World Health Report identified three broad categories of interconnected factors influencing performance: characteristics of the health workers, the health system, and the population served [9]. Performance is the result of a transactional process between health workers and their environments, as they engage with the conditions in the health system and community to provide health care according to their abilities, professional values, and personal goals [10]. This study focuses on health workers' engagement in the transactional process leading to performance, which we call “practice.”

Health workers can provide key insights into how these intersecting conditions influence their practice and how they respond to them. Increased recognition of the crippling effect of health workforce deficits in health system effectiveness has led to a growing number of studies focused on health workers in low and middle income countries. The results of studies of motivation to work in rural areas and satisfaction in these positions highlight the importance of resource availability, management support, recognition, opportunities for advancement, as well as salary levels [11–17]. 

Fewer studies have focused on how health workers respond to these conditions as they engage in their work. In the face of challenging circumstances, health workers respond with coping strategies that help them to realize their goals. Examples highlighted in the literature include strategies such as moonlighting, pilfering drugs, and informal charging, which promote personal goals to the detriment of organizational goals and performance [18–20]. Only one recent study documenting positive coping strategies that contribute to performance could be found [21].

While health worker issues have received growing attention, the context-specific nature of findings underscores the need for further assessments. There is particular need for investigation of Latin American contexts, as there are few studies of health workers from this region [22–24]. An exploratory study was conducted to gain insight into the intersecting influence of health system and community conditions on the practice of auxiliary nurses, a MLHW cadre delivering health services in a rural province of Guatemala. This paper explores auxiliary nurses' motivation and satisfaction and examines their responses to the challenging working conditions they confront in their practice. The overall aim of the study is to generate strategies for improving performance that are appropriate for addressing local conditions and can build on existing strengths. 

## 2. Methods

### 2.1. The Study Setting

In Guatemala, the health system utilizes MLHWs primarily in the form of auxiliary nurses (AN). They represent the largest cadre of health workers in the country with 18 ANs per 10,000 inhabitants in 2006, while professional nurses have a proportion of four per 10,000 and doctors, ten per 10,000 [25]. Training is one year in duration and ten years of formal schooling are required for entry to the program. Though the majority of ANs work in hospitals, they are also utilized as frontline health workers in primary and secondary care settings in rural communities where health needs are great. 

Primary care services are delivered through two types of providers: public sector health posts and nongovernmental organizations (NGOs) contracted by the government under the Coverage Extension Program (*Programa de Extensión de Cobertura*, PEC). Health posts provide coverage for a population of around 2,000 inhabitants and typically employ one to two ANs. In the PEC, NGOs are responsible for the delivery of a basic package of health services to a jurisdiction of about 10,000 inhabitants using a mobile team of health workers. The mobile team normally includes one to two ANs, one doctor or nurse, and an educator. The team makes monthly visits to the communities served in the jurisdiction catchment area. Primary care activities in both health posts and PEC include maternal and child health services, as well as attending consultations, providing educational talks, maintaining census, and epidemiological data for their catchment area. In order to gain support for health promotion activities, their work also involves communication with local leaders in the Community Development Councils as well as training and coordination with community health volunteers.

Secondary care is provided in public sector health centers, most of which are open 24 hours and provide inpatient, emergency, and maternity care as well as outpatient consultations, and maternal and child health services. Typically, health worker staff includes: a doctor or nurse who serves as district manager, one or more nurses who oversee the different departments of the health center and district health posts, a few additional doctors who work shifts, and around 15 ANs. In many sites, one AN is designated to primary care activities in the catchment area. ANs staff the different departments in the health center, including pre- and postconsultation, family planning, inpatient, and emergency. In practice, a doctor or nurse may be not available to attend patients due to position vacancies, administrative duties or when many patients are waiting. In these cases, ANs also attend patient consultations, emergencies, deliveries and manage inpatient care in secondary care settings.

The province of Alta Verapaz in northern Guatemala was selected as the site for this study because it offers rich opportunities to explore factors influencing AN practice, with positive strategic efforts alongside great difficulties. Challenges include one of the highest rates of maternal mortality in the country (207/100,000 live births) as well as the highest rate of extreme poverty (38%) [26, 27]. The majority of the rural population is monolingual in the local indigenous language with little formal education. Recent national policy to fortify health services in prioritized regions has led to scaling up health worker positions, with the creation of many new jobs in primary and secondary care services in Alta Verapaz since 2008. In Alta Verapaz as in most parts of the country, scaling up of AN positions was successful, with 97% of positions created filled, while there was 50% vacancy in the positions created for doctors and nurses [28]. However, the newly created positions are with temporary contracts and wage delays have been common. The increased number of AN positions in rural health services has been filled in part by graduates from an innovative distance education program based in the National School of Nursing in Coban, Alta Verapaz. This program aims to increase the availability of ANs who are indigenous rural residents by making education more accessible with technology and more appropriate through an adapted curriculum focused on community health and intercultural care. 

### 2.2. Data Collection

Field work was conducted from January to March, 2010 by the first author, who is a nurse by training and has established contacts with nursing schools in Guatemala through previous work. The study was supported by a committee of local stakeholders from the National Nursing School and the Regional Health Office who reviewed the study protocol, interview guide, and consent form. The director of the Regional Health Office approved the study and provided a letter to present in the districts. Field work was conducted in five districts of Alta Verapaz, in sites one to four hours from the provincial capital.

Sampling was purposive to include ANs working in both primary and secondary care as well as a balanced representation of gender and training backgrounds. A total sample of 14 ANs was achieved through 11 one-on-one interviews and one group interview with three coworkers. In the case of the group interview, the participants were coworkers with similar background, and the group dynamic provided further insight into their experience of the work environment. Characteristics of the participants are shown in Table 1. All ANs interviewed were indigenous and spoke Q'eqchi' or Poqomchi' as well as Spanish. The average age of participants was 29.5 years. Most of the ANs had temporary contracts and less than four years of experience. These characteristics were typical of the ANs present in the health services visited. 

Interviews were carried out in the work setting, except for one conducted when the AN was en route to work. Participants were informed about the purpose and focus of the interview, the voluntary nature of participation, and the confidentiality of their responses. They were provided with a written description of the research project and written consent was obtained. This process helped establish positive rapport as the participants expressed appreciation of the researcher's attention to their practice. Interviews were conducted by the first author in Spanish, and lasted from 20 to 45 minutes. Recorded interviews were then transcribed by local research assistants.

Topics in the interview guide included: motivation, their work and work environment, sources of satisfaction and frustration, sources of support, and suggestions to improve work. Additionally, contextual information was gathered through observation of ANs delivering services in health centers, health posts and PEC settings, and conversation with supervisors and community members. Field notes documenting observations and insights gained from local stakeholders were used to illustrate the context of findings from AN interviews, and provided space for beginning analysis. 

### 2.3. Data Analysis

A thematic analysis approach was used to interpret the data [29]. Analysis began with a review of the accuracy of transcriptions, followed by careful reading to become thoroughly familiar with the data set. A guide to the interview data was prepared by the first author introducing each AN, describing impressions from the interview, and observation of practice if relevant, and initial observations on points of interest were discussed by all authors. An index of themes was generated for coding the transcripts. The initial version of the theme index was based on the topics addressed in the interview guide. Analytical observations were recorded after each interview coding, and the theme index was revised to incorporate topics that emerged repeatedly and did not fit in the original themes. The themes were applied to the interviews using OpenCode 2.1 software. 

The data were then sorted into the themes, and the first author created matrices summarizing the contribution of all participants for each theme. This permitted the research team to examine and discuss the range of perceptions and experiences within each theme and across work settings. Work setting was used as the organizing category because important variations in ANs' conditions and perceptions across primary and secondary care were observed during data collection. Variation across other categories such as gender and training background were also considered. Dynamics within and connections across themes were analyzed by seeking to characterize central tendencies and examining the consistency of patterns in the experiences and attitudes expressed by workers who shared particular traits. Within the themes of motivation, satisfaction, challenges, and coping strategies, the central characteristics were community connectedness, sense of value of work, confronting resource constraints, effort of community work, and learning in practice, and these organize the presentation of results. 

## 3. Results 

### 3.1. Motivated by Community Connectedness

Participants' accounts of their motivation to work as an AN reflected a sense of connection to the people they were serving. All participants were from rural communities, though most did not work in their own village or town. However, they identified with others from rural communities, who in this province of Guatemala primarily belong to the Q'eqchi' ethnic group. They expressed that being an AN was a vocation that allowed them to help their people and provided them with an identity. Secondary care ANs were more likely to mention wanting to help their families and that they like working with people, while primary care ANs were more likely to mention helping communities. One primary care AN whose work involved travelling to distant communities with a mobile team stated:

*Even though we receive pay that is not why we go… We are helping people living in remote areas… Thank God, everything we have learned we are sharing with people at the community level (in places where) they have never had access to health services. (Participant 5, PEC)*

Among both primary and secondary care ANs, some expressions of motivation reflected the unique perspective a local health worker brings. The following quotes from two ANs illustrate how they were motivated to become an AN to address needs they had experienced themselves in their community, and also how their role provided them with identity:

*Back then, we didn't know what nursing was… Two of my sisters died one post-partum and one who was pregnant. Since we didn't know the warning signs, we just watched them there suffering for two or three days… Since we didn't have the knowledge at that time, my two sisters died. Thank God, with the achievement of studying this career, it is very important in life. (I am able to) support the communities, help the people and children. (Participant 6, Health Center)*

*(I was motivated to become an AN by) necessity. Those who worked here before were not from here… they neglected their work. They would leave on Thursday, come in late on Monday… That was how I decided to study this, and also to have a career, to be somebody in life. (Participant 4, Health Center)*

Even after describing feeling frustrated by lack of resources in her work, an AN in secondary care shared the following quote, which further illustrates how connectedness to the community as a local worker relates to motivation.

*Interviewer: Based in your experience until now, do you think you would like to continue working here?*

*AN: Yes, especially since it's my people (mi pueblo). I need to help them, because they know me and I know them. I think there is more trust between us than with another person who comes in… So I need to help my people. (Participant 7, Health Center)*

### 3.2. Satisfaction from Sense of Value of Work

ANs in primary and secondary care expressed that the nature of their work was a source of satisfaction because they were able to provide valuable services. Primary care ANs emphasized that community education and actions to prevent maternal mortality were important aspects of their work. Participant 2 expressed a clear vision of how his work activities contributed to prevention:

*For me the most important activities are firstly the immunization program because we prevent several diseases with vaccines, nutritional supplements for the children, educational talks… In PEC, we have to prevent disease so that the secondary care facilities are not overcrowded. From the community level we can prevent diseases… through education. (Participant 2, PEC)*

Secondary care ANs described that their work allowed them to help patients who came in very ill by resolving their problem or referring them. One AN said the best feeling was knowing her knowledge, skills and abilities saved a life (Participant 8, Health Center). Another area where the ANs expressed they do important work was attending deliveries. They commented on the importance of prioritizing pregnant women because two lives were at stake, and they appreciated the beauty of receiving new life. 

In general, primary care ANs expressed a stronger sense of satisfaction than those working in secondary care because their work allowed them more positive contact with communities through health promotion activities. Primary care ANs described how they derived satisfaction from being recognized in the community and feeling they had their support.

*I like what I do, being in contact with the people. It makes me happy that the people like me, they know me, I have their support… working with people in the communities, being able to help my people, that is why I feel satisfied. (Participant 7, Health Center)*

As the work of the secondary care ANs consisted in attending patients seeking services in the health center, they did not have the same opportunity for positive contact and community recognition. Instead their ability to carry out their work and resolve the patient's need was more strongly influenced by resource constraints.

While all of the ANs interviewed expressed desire to continue working in rural health settings, 12 of the 14 interviewed were working in positions with temporary contract. Several expressed that their future depended on the government and they did not know whether their positions would be renewed due to uncertainty of continued funding. 

Gender dynamics in satisfaction in different care settings merit further attention. In this sample higher satisfaction in the primary care role was confined to males. The only female AN in the primary care group expressed that she would only work in PEC a couple of more years due to the difficulties of travelling to the communities. Though she was the only AN currently employed in primary care who expressed desire to leave her position, there were four other female ANs in secondary care who had worked previously in PEC and left due to transportation difficulties. 

### 3.3. Challenges and Coping Strategies

*We fall into the error of… only seeing the bad… and we don't realize the good that one does. (Participant 8, Health Center)*

ANs described many challenging conditions which influenced their practice. However, they also shared ways they are actively coping with these conditions in their efforts to deliver services. In resource-poor settings, the constraints impeding optimal health service delivery often form the predominant impression, but as participant 8 pointed out, there were also positive aspects of AN practice that merit attention. 

#### 3.3.1. Confronting Limited Resources

In secondary care settings, lack of resources strongly influenced the ANs' practice. They described the human impact of not having the resources needed in critical situations, as well as their responses through taking initiative to resolve problems and greater attention to relationship with the patient.

Medicine shortages were common and played a large role in ANs' interaction with patients. They described the experience of giving patients prescriptions, instead of medicines, and how patients left unsatisfied. The ANs empathized with the patient perspective and expressed understanding of the effort and investment required to travel to the health center, with the expectation that they would get help. The emotional impact of patients' dissatisfaction played a strong role in their work experience, because as one participant described, “the people are dissatisfied with *you* because *you* are the one who takes responsibility (*dar la cara*) with the people.” (Participant 10, Health Center) 

Secondary care ANs also shared that the lack of resources limited their capacity to apply their knowledge and skills to resolve patient problems. In one health center, the AN related the experience of not having oxygen available to provide adequate treatment for children who came in with advanced pneumonia:

*The truth is, many children have died on us. In the case of pneumonia, they have come in already seriously ill, complicated, and sometimes they can't breathe. They have died here because we haven't been able to do anything because there is no oxygen. If we had oxygen, we would quickly put it on and refer them, and that way they would make it to the hospital. (Participant 13, Health Center)*

The ANs related these constraints to their own frustration in their work because they were not able to resolve their patients' need. 

For the most part, ANs felt that resource constraints were beyond their control. However, their motivation was also reflected in examples of ANs taking initiative to resolve problems: using their own resources to purchase small items for patients or the work setting, travelling to the Regional Health Office to try to obtain supplies, or working with community leaders to solicit support from the municipality. ANs also faced resource constraints of their own due to delayed wages which limited their ability to respond as they would have liked, because “we too are struggling to see how we are going to make it.” (Participant 12, Health Center) 

ANs also responded to these conditions through greater attention to the patient-provider relationship. They described using increased effort to communicate and achieve understanding with patients and provide good care. Their capacity to relate to the patients was strengthened by their shared language and understanding of their culture and experience. They expressed that it was important to call patients by their names, be respectful of their use of traditional medicines, and give priority to attending those who had travelled into town from the villages. The following quotes further illustrate how secondary care ANs saw themselves actively responding to the situation through their capacity to attend to patients.

*Interviewer: What makes you feel good in your work in spite of so many limitations?*

*AN: The communication with (the patients), helping them understand, explaining the reason why you can't give them what they want. Good communication… and kindness to them, that's what makes you say I did my job, I did well today. (Participant 8, Health Center)*

#### 3.3.2. Effort of Community Work

ANs in primary care also faced conditions that complicated their work, including lack of cooperation from community leaders and transportation issues. As in secondary care, their responses reflected their motivation and unique capacity to relate to the population served. 

A challenge that was unique to the primary care setting was establishing and maintaining community participation in the roles and organizations which are designed to facilitate health services coordination with the community. One AN described the initial difficulty of convincing people to participate in the Community Development Council.

*During my career I have encountered many difficulties because… since we began our work in PEC there are people who don't want to get involved in the Community Development Council… They used to say they couldn't participate because they weren't paid for their day. But thank God, we have raised awareness so that they participate and get involved in health issues, without receiving an incentive… It was really difficult… but thank God now they participate. (Participant 2, PEC)*

Another AN described the difficulty of recruiting health promoters to support his work in the health center catchment area because they were aware that volunteers in nearby communities covered by the PEC received benefits that the health center did not have the resources to offer.

*We don't have the same resources (as PEC)… (In this district) part of the population is covered by (a PEC provider). The promoters who work with me don't receive any incentives, while those with (PEC) receive monthly training, t-shirts, boots, hats, backpacks, flashlights… We don't have any of that, but I have been able to maintain them, because I have known how to manage them, how to incentivize them in other ways. If not, this would be finished. (Participant 4, Health Center)*

In both cases, the ANs described how they dealt with the perceived lack of incentives among community members through their own capacities to raise consciousness about the value of community participation and manage volunteers effectively. It is noteworthy that two ANs in a health center who had previous experience as health promoters also described how participating and sharing their nursing knowledge in their own communities was the most important way they could contribute to their progress. Their active role in community leadership outside of work seemed to enhance their sense of value in their role as ANs as well.

Primary care ANs also faced difficulty in transportation, because much of their work was in community-based health promotion activities. In the PEC program, the mobile team was sometimes provided with a vehicle or motorcycle(s). But one AN pointed out that there was not enough money for vehicle maintenance. In some cases, ANs utilized their own motorcycle to reach the communities, and paid their own fuel costs. When no vehicle was available, the ANs combined buses, rides, and walking to reach the communities where they were scheduled to provide services. Out of pocket expenditures for transport were not reimbursed. However, while these difficulties were mentioned, most of the primary care ANs did not seem to be demoralized by this aspect of their work. They expressed more satisfaction derived from their contact with the communities. 

However, this pattern of greater satisfaction derived through primary care community work did not appear to hold for female ANs interviewed. Feedback from stakeholders also indicated that female ANs from rural communities were often more reticent and could experience greater difficulty gaining recognition with community leaders, due to traditional gender roles particularly in remote areas.

#### 3.3.3. Learning in Practice

Knowledge limitations constrained ANs in certain areas of their work, such as diagnosing patients, knowing which treatment to prescribe, filling in paperwork correctly, and attending deliveries. However, they also described strategies to improve their knowledge through learning from other health professionals in practice.

ANs from all settings and training backgrounds expressed that education is theory, implying that in training they acquired knowledge from books but lacked opportunity to practice. They shared that during training in the clinical setting, there was often a large group of students and it was difficult to get hands-on experience. ANs working in understaffed health centers sometimes mentioned that some of their work activities were outside of their scope of practice, but both ANs and supervisors expressed that they should not leave patients unattended or send patients away because there was no doctor. Given the one-year duration of training, shortage of health professionals, and limited time of experience of many of the newly contracted ANs, the structural conditions contributing to this constraint were evident. 

Several ANs who were recent graduates of the distance education program expressed an attitude of “learning in practice” that seemed to help them cope with their knowledge limitations. One AN shared that he always asked questions during clinical training. When he did not understand, he would “stop a doctor, any doctor and ask a question.” Other recent graduates working in a health center said that they had gotten where they were by not standing by passively. They put themselves in the action so they could learn and improve.

*That's why we have learned something in life—if we weren't this way, we wouldn't have learned anything… we have never let things pass, we always get ourselves in. (Participant 13, Health Center)*

On the job, they had improved by learning with the doctors, taking advantage of opportunities to observe how they attend deliveries, and by receiving orientation about classes of disease and their treatment. Learning was facilitated by positive relationships with doctors. One medical director stated that he trained the ANs to do his job because he was only one person and sometimes he was not there or there were too many patients. Nurse supervisors also contributed to an organizational culture that promoted team work. A health center AN recounted that their supervisor told them that if they were going to work well, they had to work as a team and help and support each other. Other strategies to confront knowledge limitations included: seeking advice from more experienced ANs and nurse supervisor in person and by phone and consulting the Standards of Practice manual. Learning was described as a source of satisfaction in work and continued learning was frequently named as what was most needed to help them work more effectively in their role as an AN. 

## 4. Discussion 

The findings of this study illustrate interconnections between motivation, satisfaction, and health workers' practice in a rural Latin American setting. The ANs described constraints to service delivery common in low resource settings: shortages of medicines and equipment, travel to distant communities, difficulties of community participation, and knowledge limitations. The coping strategies they used to respond to these constraints in practice reflected their motivation through connection to the community they were serving and satisfaction from a sense of the value of their work. These responses provide insight into locally-developed strategies that strengthen the health worker's relationship to the health system as well as the community. 

The health worker characteristic of “community connectedness” was central to motivation in this rural Guatemalan setting. ANs interviewed expressed desire to serve their people and address health needs of their community and families. The value of residing in the community was contrasted with the historical experience of health workers coming from outside. Other studies have also highlighted the importance of intrinsic motivation among rural health workers. They describe professional ethos, religious conviction, and desire to help the poor as factors that should be built on in efforts to improve performance [12, 30, 31]. 

Satisfaction was derived from the sense that their work was addressing important health needs. ANs working in primary care settings described that their satisfaction with their work was reinforced by recognition from communities, particularly in their health promotion activities. The experience of the ANs working in secondary care settings reflected more tension between satisfaction with the value of their work and frustration due to resource constraints that restricted their ability to resolve patient problems. But all ANs interviewed reported that they planned to continue working in the health services, which is an indicator of satisfaction. This resonates with findings on Brazilian health agents: “When agents talked about why they liked their jobs, the subject of respect from clients and “my community” often dominated the conversation” (cited in [32]). Franco et al. describe this as social embeddedness and contend that it affects health worker motivation to provide good service [32]. The findings of this study provide further evidence of the far-reaching influence connectedness to the population served can have on practice. 

Health worker motivation is typically seen in the literature through the human resource management (HRM) lens and defined as “the willingness to exert and maintain an effort towards organizational goals” [32]. Studies seek to identify determinants of health worker motivation so that performance management strategies may be customized [13, 14, 17]. In this view, the lack of health worker motivation is the problem impeding performance and enhancing their motivation is the solution. The findings of this study suggest that in some contexts motivation may not be the problem. Instead, the perspectives shared by ANs raise the question whether the health system organization is giving them the support they need to enable them to meet their goals arising through their own experience of health needs in their community. Fritzen has also pointed out that performance management strategies are difficult to apply in public services of developing countries where essential pre-requisites, including availability of drugs, equipment, and transport as well as living wages for workers, are often missing [33]. 

The ANs' motivation and satisfaction led them to utilize coping strategies that reflected that their personal goals were closely related to organizational goals. These strategies indicate directions for strengthening health workers' relationship to the health system and the community. The displayed coping strategy of learning with colleagues in practice is similar to existing recommendations to support performance through promoting lifelong learning and team work in an enabling work environment [9]. ANs taking initiative to solve resource constraints also resembles the desired outcome of suggested strategies for preparing health workers to adapt to challenges through problem-based learning and providing conditions for agency [7, 9]. These strategies indicate directions for strengthening health workers' integration with the health system, which are in line with existing recommendations for bringing their practice in line with standards of care and enabling them to adapt to changing conditions. 

The coping strategies exhibited by ANs of greater attention to the patient relationship and building collaborative relationships with community leaders indicate directions for strengthening health worker relationship to the community which have received little attention in the literature. These strategies built on ANs' shared cultural and linguistic background with the population served and illustrated how community connectedness translated to a unique capacity for responsiveness in their practice. A recent study of rural health workers in Papua New Guinea describes how, even when health workers are not locals, efforts to build engagement with the community contribute to motivation and performance through the increased acceptability of services [34]. In order to strengthen the health worker's position as a bridge between the community and the health system, greater emphasis is needed on community-oriented strategies for a more balanced approach to improving performance.

These findings differ from other examples of coping strategies in the literature, which focus on health workers reacting to adverse conditions through actions that help them to attain personal goals that are not in line with organizational goals [32]. It is possible that these findings are due to differences in the severity of the adversity in the conditions faced in this setting compared to others, or perhaps reflect the relatively short time of experience of many of the participants. But it also seems important that research on rural health care is usually conducted from a “deficit-describing” approach [33]. Lindelow & Serneels justify their focus on performance problems and the exclusion of the positive experiences of health workers and users as the “most direct method to understanding current issues and identifying potential solutions” [18]. Positive coping mechanisms and existing strengths also deserve attention as they can provide insight into local strategies that can be built on in developing context-appropriate support. Gross et al. suggest focusing on “workhood assets” as a means of exploring health workers' capacity to cope, adapt and actively respond to constraints to performance in resource-poor settings [21]. 

The findings of this study also brought to light a gender difference among health workers in this setting. Male ANs were more prevalent in primary care in this setting, while female ANs expressed that the burden of travel to distant communities was too great, particularly when they also had family obligations. This contrasts with other MLHW cadres such as the Lady Health Workers in Pakistan and Health Extension Workers in Ethiopia which exclusively enroll women to provide community level primary care. These countries' utilization of female health workers is based on their acceptability and cultural sensitivity in women's health issues. However, this study suggested that gender-related characteristics also posed a constraint for women in the primary care role, limiting their mobility. 

This study aimed to generate insight into the practice of MLHWs in rural Guatemala and strategies for supporting their performance through the perspective of front-line workers. However, the results should be considered in light of the following limitations. Due to access issues and time limitations, the most remote workers were not represented in this sample. Language barrier also may have limited full understanding in the interviews, as Spanish was the second language of participants and the interviewer. Analysis of variation across health worker characteristics focused primarily on differences between primary and secondary care settings, even though these differences also coincided with gender in this sample. This decision was based on the observation during field work that the primary care role offered greater opportunity for self-realization and positive community contact. It is possible that the importance of gender in explaining variation across work settings has been underestimated.

## 5. Conclusions 

The AN cadre exhibits the characteristics that make MLHWs a strategic focus for strengthening health systems. They play a prominent role in service delivery in rural areas of Guatemala with great health needs. Recruitment of rural residents is facilitated by low entry education requirement and distance education modalities. Successful scaling up of AN positions in priority areas of Guatemala in 2008 contrasted with high vacancy rates in new positions for professionals. Current international priorities emphasize efforts to improve rural health services through incentive-based strategies to attract and retain professionals who are not motivated to work in rural areas [35]. Given the difficulty of attracting professional to rural facilities, improving support for the health workers who are already there is a logical complementary step to strengthen performance of the health system [16]. 

This study highlighted existing strengths of individuals from a MLHW cadre who were motivated by community connectedness and employed a range of coping strategies in their practice in the face of difficult circumstances. Policies to build on these strengths should focus on mechanisms that make midlevel as well as professional health worker training accessible to rural residents, such as targeted recruitment, housing allowances for study, and distance education. Education level required for entry is also an important factor in accessibility. Continued support for MLHWs in practice should provide them the opportunity to develop their capacity as problem solvers, strengthen their skills through mentoring relationships, and increase lines of communication with professionals for consultation. Finally, the practice of health workers in rural areas should be strengthened through efforts to fortify their relationship to the community, such as collaborative work with leaders and volunteers and greater attention to the provider-patient relationship. These recommendations indicate actions to make effective and efficient use of existing human resources and enhance their capacity to deliver health services to underserved populations in rural Guatemala and other low-resource settings.

## Figures and Tables

**Table 1:** Characteristics of auxiliary nurses interviewed.

Participant	Age	M/F	Work site	Level of care	Experience (yrs)	Previous work	Contract	Training
1	36	M	HP	1°	1.5	—	Temporary	DE
2	29	M	PEC	1°	10	—	Temporary	NSN
3	25	F	PEC	1°	1.5	—	Temporary	DE
4	37	M	HC	1°	13	—	Permanent	NSN
5	34	M	PEC	1°	4	CHW	Temporary	DE
6	28	M	HC	1°	1.5	CHW	Temporary	DE
7	21	F	HC	2°	2	Hospital	Temporary	NSN
8	41	F	HC	2°	14.5	—	Permanent	NSN
9	25	F	HC	2°	4	PEC	Temporary	NSN
10	24	F	HC	2°	3	PEC	Temporary	NSN
11	26	F	HC	2°	1.5	PEC	Temporary	DE
12	29	F	HC	2°	1.5	PEC	Temporary	DE
13	24	M	HC	2°	1.5	—	Temporary	DE
14	34	M	HC	2°	1.5	CHW	Temporary	DE

Average	29.5				4.4			

HP: health post; PEC: coverage extension program; HC: health center; CHW: community health worker; NSN: National School of Nursing; DE: distance education.

## List of Abbreviations

AN: Auxiliary nurse
MLHW: Midlevel health worker
NGO: Nongovernmental organization
PEC: Programa de Extensión de Cobertura (Coverage Extension Program).

## References

[1] Lehmann U Mid-level health workers. The state of the evidence on programmes, activities, costs and impact on health outcomes. A literature review.

[2] Dovlo DY (2004). Using mid-level cadres as substitutes for internationally mobile health professionals in Africa. A desk review. *Human Resources for Health*.

[3] Crisp N, Gawanas B, Sharp I (2008). Training the health workforce: scaling up, saving lives. *The Lancet*.

[4] Pereira C (2010). *Task-shifting of major surgery to midlevel providers of health care in Mozambique and Tanzania a solution to the crisis in human resources to enhance maternal and neonatal survival [Ph.D. thesis]*.

[5] Bangdiwala SI, Fonn S, Okoye O, Tollman S (2010). Workforce resources for health in developing countries. *Public Health Reviews*.

[6] Fonn S, Ray S, Blaauw D (2011). Innovation to improve health care provision and health systems in sub-saharan africa-promoting agency in mid-level workers and district managers. *Global Public Health*.

[7] Mwita N The changing role of mid level and community health workers in the health system in Africa.

[8] Songstad NG, Rekdal OB, Massay DA, Blystad A (2011). Perceived unfairness in working conditions: the case of public health services in Tanzania. *BMC Health Services Research*.

[9] World Health Organization The World Health Report 2006: Working Together for Health.

[10] Rowe AK, de Savigny D, Lanata CF, Victora CG (2005). How can we achieve and maintain high-quality performance of health workers in low-resource settings?. *The Lancet*.

[11] Lehmann U, Dieleman M, Martineau T (2008). Staffing remote rural areas in middle- and low-income countries: a literature review of attraction and retention. *BMC Health Services Research*.

[12] Mathauer I, Imhoff I (2006). Health worker motivation in Africa: the role of non-financial incentives and human resource management tools. *Human Resources for Health*.

[13] Dieleman M, Cuong PV, Anh LV, Martineau T (2003). Identifying factors for job motivation of rural health workers in North Viet Nam. *Human Resources for Health*.

[14] Dieleman M, Toonen J, Touré H, Martineau T (2006). The match between motivation and performance management of health sector workers in Mali. *Human Resources for Health*.

[15] McAuliffe E, Bowie C, Manafa O (2009). Measuring and managing the work environment of the mid-level provider—the neglected human resource. *Human Resources for Health*.

[16] Bradley S, McAuliffe E (2009). Mid-level providers in emergency obstetric and newborn health care: factors affecting their performance and retention within the Malawian health system. *Human Resources for Health*.

[17] Willis-Shattuck M, Bidwell P, Thomas S, Wyness L, Blaauw D, Ditlopo P (2008). Motivation and retention of health workers in developing countries: a systematic review. *BMC Health Services Research*.

[18] Lindelow M, Serneels P (2006). The performance of health workers in Ethiopia: results from qualitative research. *Social Science and Medicine*.

[19] Segall M (2000). From cooperation to competition in national health systems- and back?: impact on professional ethics and quality of care. *The International Journal of Health Planning and Management*.

[20] Van Lerberghe W, Conceição C, Van Damme W, Ferrinho P (2002). When staff is underpaid: dealing with the individual coping strategies of health personnel. *Bulletin of the World Health Organization*.

[21] Gross K, Pfeiffer C, Obrist B (2012). ‘Workhood’-a useful concept for the analysis of health workers' resources? an evaluation from Tanzania. *BMC Health Services Research*.

[22] García-Prado A, Chawla M (2006). The impact of hospital management reforms on absenteeism in Costa Rica. *Health Policy and Planning*.

[23] Hermida J, Robalino ME (2002). Increasing compliance with maternal and child care quality standards in Ecuador. *International Journal for Quality in Health Care*.

[24] Svitone EC, Garfield R, Vasconcelos MI, Craveiro VA (2000). Primary health care lessons from the Northeast of Brazil: The Agentes de Saude Program. *Revista Panamericana de Salud Pública*.

[25] Estrada Galindo G, Slowing Umaña K Síntesis?: Sistema de salud en Guatemala?: *¿*hacia dónde vamos?.

[26] Ministerio de Salud Pública y Asistencia Social, Segeplan   Encuesta nacional de mortalidad materna.

[27] Instituto Nacional de Estadística   ENCOVI: Encuesta nacional de condiciones de vida.

[28] Organización Panamericana de Salud Estudio: Información sobre recursos humanos de salud en Guatemala.

[29] Lewis J, Spencer L, O’Connor W, Ritchie J, Lewis J (2003). Carrying out qualitative analysis. *Qualitative Research Practice: A Guide for Social Science Students and Researchers*.

[30] Manongi RN, Marchant TC, Bygbjerg IC (2006). Improving motivation among primary health care workers in Tanzania: a health worker perspective. *Human Resources for Health*.

[31] Serneels P, Montalvo JG, Pettersson G, Lievens T, Buterae JD, Kidanu A (2010). Who wants to work in a rural health post? the role of intrinsic motivation, rural background and faith-based institutions in Ethiopia and Rwanda. *Bulletin of the World Health Organization*.

[32] Franco LM, Bennett S, Kanfer R (2002). Health sector reform and public sector health worker motivation: a conceptual framework. *Social Science and Medicine*.

[33] Fritzen SA (2007). Strategic management of the health workforce in developing countries: what have we learned?. *Human Resources for Health*.

[34] Razee H, Whittaker M, Jayasuriya R, Yap L, Brentnall L (2012). Listening to the rural health workers in Papua New Guinea—the social factors that influence their motivation to work. *Social Science & Medicine*.

[35] Ranson MK, Chopra M, Atkins S, Dal Poz MR, Bennett S (2010). Priorities for research into human resources for health in low- and middle-income countries. *Bulletin of the World Health Organization*.

